# Knowledge on Bone Banking among Participants in an Orthopaedic Conference: A Preliminary Survey

**DOI:** 10.5704/MOJ.1707.004

**Published:** 2017-07

**Authors:** S Mohd, N Yusof, S Ramalingam, WM Ng, A Mansor

**Affiliations:** Department of Orthopaedic Surgery (NOCERAL), Faculty of Medicine, University of Malaya, Kuala Lumpur, Malaysia

**Keywords:** awareness, bone banking, bone graft, knowledge, orthopaedic community

## Abstract

Despite increasing use of bone graft in Malaysia, there was still lack of data to quantify knowledge level on bone banking among orthopaedic community who are involved in transplantation related work. Therefore, a survey on awareness in tissue banking specifically bone banking, usage and choice of bone grafts was conducted. From 80 respondents, 82.5% were aware about tissue banking however only 12.5% knew of the existence of tissue banks in Malaysia. Femoral head was the bone allograft most often used as a substitute to autograft. Only 34.8% respondents preferred irradiated bone grafts whilst 46.9% preferred nonirradiated, indicating the need to educate the importance of radiation for sterilising tissues. Exhibition was the most preferred medium for awareness programme to disseminate information about bone banking in the orthopaedic community. The professional awareness is necessary to increase the knowledge on the use of bone graft, hence to increase bone transplantation for musculoskeletal surgeries in the country.

## Introduction

Bone grafting procedure has already been practised in orthopaedic transplantation for more than a hundred years^1^. Autograft is considered the gold standard; however, due to the limited availability and amount of bone and donor site morbidity, the use of allograft was introduced in the early 1980s’. Bone allograft utilised as a substitute in orthopaedic procedures serves three main functions: providing mechanical support, roadway for blood vessel and cell ingrowth^2^ and supplying growth factors. Bone allografts have been transplanted into more than five million cases in the United States alone. Nevertheless, bone banking is not well known among the orthopaedic surgeons despite the increasing usage of allografts in orthopaedic procedures^3^. In Malaysia, 273 musculoskeletal tissue grafts were supplied to various hospitals by three tissue banks between 2011 and 2013^4^. The number of distributed grafts was comparatively smaller than the number of orthopaedic procedures requiring bone performed in Malaysia. Approximately there were 4,000-5,000 arthroplasty cases per year nationwide (unreported data). University Malaya Medical Centre (UMMC) as the tertiary referral centre for complex arthroplasty cases registered 16 out of 600 cases requiring bone allograft in 2016. We therefore conducted a survey to determine the level of bone banking knowledge in the orthopaedic community who participated in an orthopaedic conference. The questionnaire was designed to capture the usage and actual demands of bone allografts. Their preference of methodology for awareness programme was also explored.

## Materials and Methods

**Fig. 1:**
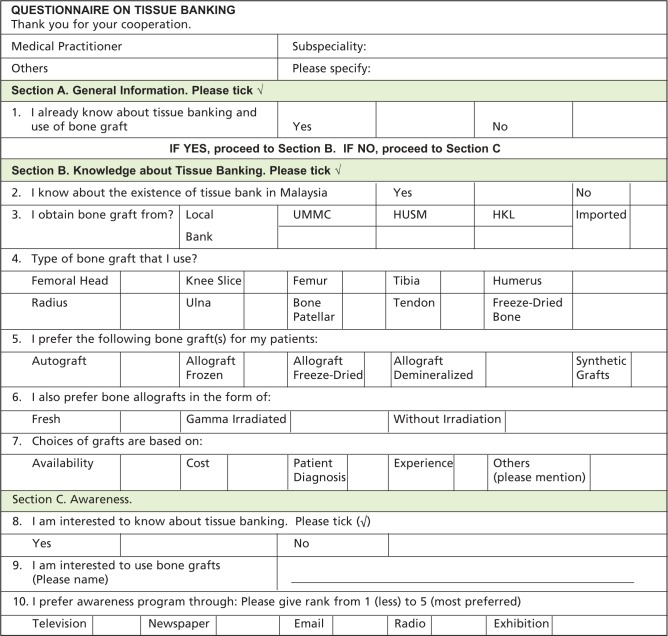
The survey form.

Self-designed and semi-structured questionnaire was created to capture flexibility of data. The questionnaire was validated and the reliability analysis attained the Cronbach’s Alpha value of > 0.70. Data was collected during the 44th Malaysian Orthopaedic Association (MOA) Annual General Meeting (AGM)/Annual Scientific Meeting (ASM) from 26 to 28 May 2014. This is a preliminary survey targeting on orthopaedic community, specifically orthopaedic surgeons, nurses and paramedics who were involved in tissue transplantation. A convenience sampling method with total of 100 questionnaires were successfully distributed among participants. The questionnaires were distributed in our research centre booth (NOCERAL) whereby there was information from various units including Bone Bank. The respondents who visited the booth were verbally screened whether they knew or were interested to know about bone banking before their participation in this survey. Respondents were required to fill up the survey consisting of ten questions, which were divided into three sections (Fig. 1).

## Results

**Fig. 2:**
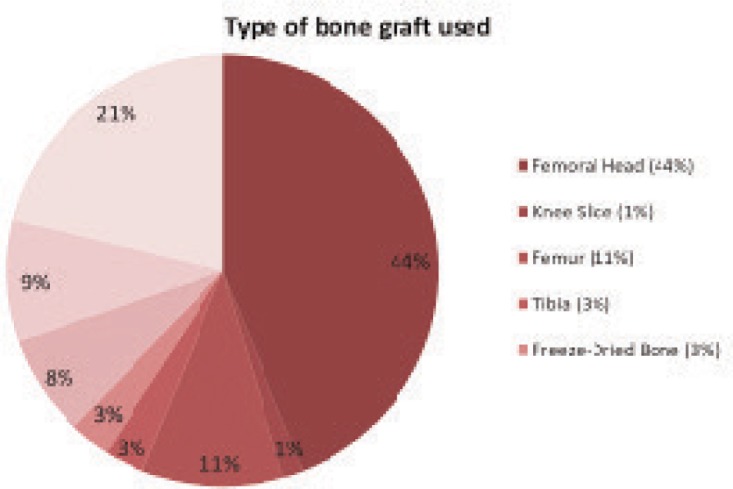
Type of bone graft used. Femoral Head (FH): 29; Femur: 7; all: 6; combination of any bone graft: 5; Tibia: 2; Freeze-Dried Bone: 2, Knee Slice: 1; no answer: 21 (n: 66).

Only 80 of 100 questionnaires distributed during the MOA Annual Scientific Meeting were returned. The 80 respondents comprised of 60% medical doctors and 40% others (nurses and paramedics). General knowledge on tissue banking was considerably high with 82.5% (66 respondents) answered Section A and B. Among the 66 respondents who had prior knowledge on tissue banking, 74.2% were aware of the existence of tissue banks in Malaysia namely HUSM Tissue Bank (27.3%), HKL Bone Bank (13.6%) and UMMC Bone Bank (9.1%); while 10.6% knew two out of the three banks, 15.2% knew all the three banks and 24.2% did not know any of the banks. The surgeons obtained the bone grafts from HUSM Tissue Bank (36.4%), UMMC Bone Bank (22.7%) and HKL (1.5%). Some obtained bone allografts from both HUSM Tissue Bank and UMMC Bone Bank (3.1%) and some used imported allografts (4.5%). The rest who did not answer (31.8%) were nurses and paramedics. As presented in Figure 2, femoral head was the most commonly used bone graft (44%), followed by cadaveric femur (11%), use of any two of the bone grafts (8%) and use of all types of bone (9%). A few answered that they used freeze-dried bones (3%), tibia (3%) and knee slices (1%). The 21% of respondents who did not answer the question were nurses or paramedics who were not involved in decision making in choosing the grafts.

**Fig. 3:**
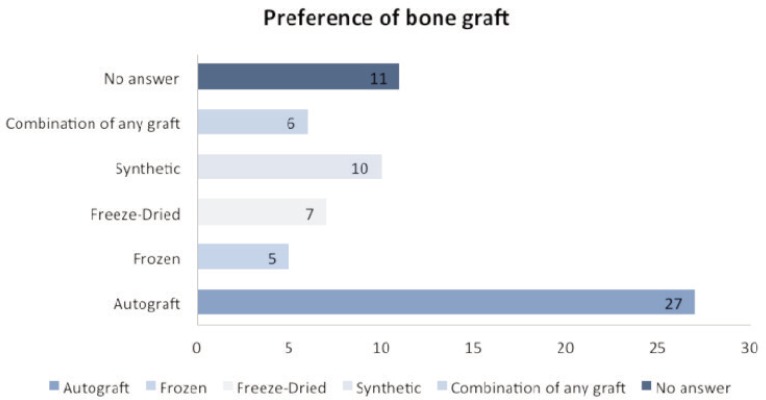
Preference of bone graft. Autograft: 27; Synthetic bone: 10; Freeze-Dried: 7; Frozen: 5; Combination of any graft: 6; No answer: 11 (n: 66).

On the question about the preference of bone grafts (Fig. 3), as expected a high percentage of the respondents (40.9%) preferred autografts. Only 18.2% of the respondents would use allografts either freeze-dried (10.6%) or frozen bones (7.6%) and almost equal percentage of the respondents (15.2%) would use imported synthetic grafts. Some would use more than one type of graft including synthetic grafts (9%) while 16.7% did not answer. With regards to sterilisation, 46.9% preferred non-irradiated bone graft both fresh and processed bones; while 34.8% preferred irradiated bone grafts and 18.3% did not answer.

**Fig. 4:**
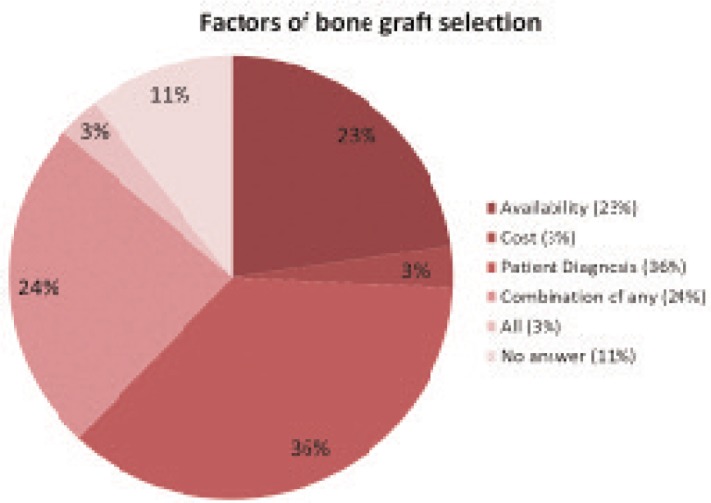
Factors of bone graft selection. Patient Diagnosis: 24; Availability: 15; Cost: 2; Combination of any: 16; All: 2; No answer: 7 (n: 66).

The selection of bone grafts was primarily based on patient’s indication and diagnosis (36%) followed by availability of allografts (23%), combination of several factors (24%) and all listed factors (3%). The cost of the graft did not affect the decision (3%) whilst 11% did not answer (Fig. 4).

On the question about awareness programme, majority of the respondents (86.3%) were willing to learn more about tissue banking; however, a few were not interested to know (2.5%) and some were not sure (11.2%). They preferred the awareness programme to be conducted through exhibition (36.3%) followed by through email (25%) and television (21.2%). The rest of the respondents would still prefer the conventional methods through radio (2.5%) and newspaper (3.8%), while 11.2% did not answer.

## Discussion

The survey was conducted during the 44th annual meeting of Malaysian Orthopaedic Association, a prestigious event attended by 1143 participants from all over Malaysia, of whom 465 were orthopaedic surgeons. The meeting provided a platform for the surgeons to update knowledge on surgical techniques and to keep abreast with the current research and development in orthopaedics. The event provided the right platform to gauge the knowledge of the community on tissue banking while they were away from their busy clinical duties. The questionnaires were distributed selectively to those we knew or expected to be involved in bone transplantation.

The study managed to get feedback of the distributed questionnaires from 80% of the participants. The non-responding participants were probably too busy to fill up the survey form while in the midst of attending the conference and meeting. Besides the orthopaedic surgeons, 40% of the respondents were nurses and paramedics. This was the reason why some questions in the Section B of the questionnaire were not answered.

A high percentage of respondents knew about bone banking indicating they have knowledge on the use of musculoskeletal tissues in orthopaedic surgeries. They knew about HUSM Tissue Bank in Kubang Kerian, Kelantan, more than the two bone banks in Kuala Lumpur, namely at University Malaya Medical Centre (UMMC) and Hospital Kuala Lumpur (HKL). The HUSM Tissue Bank has been the main supplier of allografts and xenografts since it was officially established in 1994 and is the only tissue bank in the country. The bank is ISO certified with GMP as well as syariah compliance^5^. The bank provides bone grafts in various forms: fresh frozen, freeze-dried and demineralised bone grafts. The bone banks in UMMC and HKL mainly provide fresh frozen bone allografts for in-house use and local surgeons. The UMMC Bone Bank has been collecting femoral heads from arthroplasty and trauma cases since 2000 and recently the bank has been able to provide femoral heads and knee slices to other local hospitals^6^. HKL Bone Bank, established in 1998, has been procuring cadaveric bones with coordination of the National Transplant Resource Centre (NTRC), Ministry of Health (MOH), and providing large bone allografts to mainly the government hospital surgeons.

The results indicated that femoral head was the most commonly used type of bone allograft (44%). The bone is mainly needed in arthroplasty cases as space filler in the form of milled or chipped bones. Some surgeons used cadaveric long bones from lower limb namely, tibia and femur (14%) and processed bone graft (11%), in reconstruction and spinal surgeries, respectively.

The choice of bone grafts was mostly determined by patient’s indication based on diagnosis, followed by availability of the required bones. Cost was not the main factor influencing selection of bone grafts. The local bone banks seemed to be able to continuously supply bone grafts for patients at any desired time of surgeries and preferably at a reasonable cost^7^. For example, UMMC Bone Bank imposes service charge at RM 750 per femoral head, comparatively lower than the imported femoral head available on average at RM 4,000.

In Malaysia, most of bone grafts are irradiated at 25kGy for sterilisation and minimizing or avoiding the risk of disease transmission. Radiation has been recommended by standards issued by Asia Pacific Association of Surgical Tissue Banking (APASTB) and American Association of Tissue Banks (AATB). Currently radiation is the best choice compared to heat treatment and ethylene oxide gas, which cause damage to biological components and leave toxic residues, respectively. However, the survey revealed that 46.9% of the respondents preferred non-irradiated bone graft. Although fresh frozen bone is associated with the best long term results^8^ it has high risk of disease transmission^9^. In this case, the local tissue banks have to convince the users that radiation does not affect biomechanical properties of specifically weight bearing bones when irradiated in deep frozen state. According to Hamer *et al*^10^ changes in the bone mechanical strength could only be observed at radiation doses higher than 60kGy. Therefore, limited knowledge on radiation effects may become an obstacle for bone bank in producing safe grafts^11^. Organising professional awareness programme and technical workshop on radiation technology and effects of radiation on biomechanical strength of bones seemed to be necessary. Continuous awareness programme on bone banking is aimed to update knowledge among members of the orthopaedic community and to educate especially the newcomers. Knowledgeable surgeons, nurses and paramedics will then assist in educating the public to make them aware about tissue donation in general and the roles of tissue banks.

The growing need of tissues such as bones for transplantation would require new strategies to enhance potential donors^12^. Our study indicated that awareness programme through exhibition had the best preference by the orthopaedic community. Similar finding was obtained by the first survey on tissue banking conducted among the Malaysian public^13^. On the contrary, printed and electronic media were more popular than exhibition for organ donation awareness programme^14^. Through exhibitions, the orthopaedic community could easily get information through direct conversation and technical discussions. However dissemination of information through digital media like email and television cannot be ignored. The UMMC Bone Bank will continue putting up exhibition booths at relevant events such as annual conferences or meetings organised by the Malaysian Society of Transplantation (MST) and the Malaysian Orthopaedic Association (MOA).

Professional training seemed to be an effective way for creating awareness and attitude of doctors and nurses to tissue donation and banking^15^. Therefore, the Malaysian Association of Cell and Tissue Banking (MACTB) conducted workshops on bone and bone and eye procurement in 2013 and 2015, respectively. The Procurement Workshop will be organised every two years. Besides procurement, awareness on the use of bone allograft is crucial whereby the university hospital doctors and specialists can play a role to promote bone transplantation^16^. In many other countries, awareness on tissue transplantation is still far behind the organ transplantation^17^. Tissue and Bone Banks in Malaysia therefore need to be actively involved in the public and professional awareness programmes organised by the NTRC in promoting tissue donation and tissue banking. More frequent educational seminars and workshops will be conducted.

## Conclusion

The survey provided useful information in identifying bone requirements by the local surgeons. The UMMC Bone Bank will improve its services to fulfil demands for the bone grafts to be used in transplantation. The survey managed to provide a meaningful feedback that will assist in designing professional awareness programmes to continuously disseminate knowledge to orthopaedic community and to share information on bone banking activities including bone retrieval, processing, sterilisation and storage. Professional training on the use of bone graft for young surgeons is deemed necessary as it may lead to increase in bone transplantation in the country. Nevertheless, the present study was limited to the participants of a single conference and would not represent overall feedback from the orthopaedic community in Malaysia. Hence, surveys with larger sample size need to be conducted to determine the level of knowledge on bone banking among orthopaedic community specifically those involved in bone transplantation in the country.
